# Clinical efficacy of oxidized regenerated cellulose powder in perioperative blood management in direct anterior total hip arthroplasty

**DOI:** 10.1051/sicotj/2025036

**Published:** 2025-07-16

**Authors:** Taizo Kaneko, Kentaro Hayakawa, Tsuyoshi Miyazaki

**Affiliations:** Department of Orthopaedic Surgery, Tokyo Metropolitan Institute for Geriatrics and Gerontology Sakae-cho 35-2, Itabashi-ku Tokyo 173-0015 Japan

**Keywords:** Oxidized regenerated cellulose powder, Blood loss, Hidden blood loss, Total hip arthroplasty, Direct anterior approach

## Abstract

*Background*: Perioperative blood loss remains a challenge in total hip arthroplasty (THA). Although tranexamic acid (TXA) is widely used for hemostasis, the efficacy of oxidized regenerated cellulose (ORC) powder as an adjunct in blood management for THA via the direct anterior approach (DAA) remains underexplored. This study aimed to evaluate the effects of ORC powder on perioperative blood loss, hematological parameters, and clinical outcomes in direct anterior THA. *Methods*: A total of 133 patients who underwent primary THA via the DAA were enrolled in the study. The patients were divided into two groups: the ORC powder group (combination of ORC powder and topical TXA, *n* = 53) and the control group (topical TXA alone, *n* = 80). The demographic and clinical information, operative time, intraoperative bleeding volume, estimated total blood loss (eTBL), hidden blood loss (HBL), trends in hemoglobin, hematocrit, postoperative pain scores using a numeric rating scale (NRS), and adverse events were analyzed. Clinical outcomes were assessed using the Japanese Orthopedic Association score. *Results*: The ORC powder group had significantly lower eTBL (679.1 ± 230.1 mL vs. 875.8 ± 292.9 mL, *p* < 0.0001) and HBL (424.1 ± 194.5 mL vs. 558.6 ± 264.2 mL, *p* = 0.002). Postoperative pain scores at postoperative day 7 were lower in the ORC powder group (1.9 ± 1.6 vs. 2.9 ± 2.2, *p* = 0.009). The clinical outcomes were excellent, and no significant differences were observed in complication rates between the groups. *Conclusion*: ORC powder effectively reduced perioperative blood loss in THA via the DAA without increasing complication rates. ORC powder has the potential to be a valuable adjunct in optimizing blood management strategies in THA.

## Introduction

Total Hip Arthroplasty (THA) is a widely performed surgical procedure aimed at alleviating pain and restoring mobility in patients with degenerative hip conditions [1]. Globally, the number of THA procedures continues to rise, exceeding 1.4 million annually, and is expected to double by 2040, owing to the aging population and increasing life expectancy [2].

Despite the advancements in surgical techniques, perioperative bleeding in THA remains a problem that must be overcome. Previous studies indicate that perioperative blood loss in THA can surpass 1,500 mL, leading to hemoglobin reductions in the immediate postoperative period [3–5]. Excessive perioperative blood has been linked to prolonged hospitalization, an increased risk of postoperative infections, and prolonged postoperative pain due to hematoma formation [6–8]. Therefore, effective perioperative blood management is crucial in THA.

The direct anterior approach (DAA) is a minimally invasive surgical technique that has gained popularity owing to its muscle-sparing nature and the potential for faster recovery than other approaches [9]. However, it presents unique challenges, particularly in controlling bleeding from the soft tissues and periarticular structures.

Hemostatic agents, such as tranexamic acid (TXA) [5, 10, 11] and oxidized regenerated cellulose (ORC) [12–14] have been introduced as adjunctive measures to minimize bleeding during various surgical procedures. SURGICEL^®^ Powder, an ORC-based hemostatic agent, facilitates localized clot formation and is valued for its ease of application and rapid hemostatic action [14]. Its ease of application and rapid action make it a valuable tool in surgical practice, particularly for procedures with a high risk of bleeding. Notably, ORC powder has demonstrated efficacy in controlling blood loss in various surgical settings, including cardiovascular and general surgery [12, 15]. Its powder formulation is particularly advantageous for managing bleeding at inaccessible sites, making it a promising candidate for THA, where bleeding control is often challenging. Despite these advantages, the potential efficacy of ORC powder in managing perioperative blood loss in patients who underwent THA via the DAA remains underexplored.

The primary aim of this retrospective study was to evaluate the potential efficacy of ORC powder in managing perioperative blood loss in THA via the DAA. Secondary objectives included assessing its impact on hematological parameters, clinical recovery outcomes, postoperative pain reduction, and adverse events.

## Materials and methods

### Patient population

Data were collected from patients who underwent primary THA via DAA for hip osteoarthritis between July 2020 and April 2025 at our hospital. Patients with THA due to femoral neck fractures or a history of hematological or bleeding disorders were excluded. The use of ORC powder in all THA procedures commenced in June 2024 following its approval for use at our institution. Patient data were retrieved from the electronic medical records system. The medical records of the remaining 133 patients were reviewed to obtain demographic and clinical information including age at surgery, sex, body mass index, American Society of Anesthesiologists Physical Status, operative time, and intraoperative bleeding volume. Laboratory data such as hemoglobin (Hb), hematocrit (Hct), and C-reactive protein (CRP) levels measured preoperatively and on postoperative days (POD) 1 and 7 were reviewed. Adverse events, including superficial or deep infections and venous thromboembolism, were evaluated. Autologous blood deposition was performed in patients with a preoperative Hb of 11 or higher, without serious cardiac disease, and with a request from the patient for autologous blood storage. In cases where autologous blood deposition was performed, 400 cc of blood was collected at least two weeks prior to surgery. In cases where autologous blood was collected, the blood was transfused on the day after surgery or the following day.

All patients were provided informed consent, and the study protocol was approved by the institutional review board of the authors’ affiliated institution (R24-094).

### Outcome measures

The primary outcome was perioperative blood management evaluated using estimated total blood loss (eTBL) and hidden blood loss (HBL). Secondary outcomes included trends in Hb, Hct, and CRP levels, as well as the incidence of adverse events, including superficial or deep infection, and venous thromboembolism (deep vein thrombosis, symptomatic pulmonary embolism). Postoperative pain was evaluated using a numeric rating scale (NRS) [16], were evaluated. Clinical outcomes were assessed using the Japanese Orthopedic Association (JOA) score [17]. This evaluation system consists of a 100-point scale comprising four subcategories, i.e., pain around the hip joint (40 points), range of motion (20 points), walking ability (20 points), and activity of daily living (20 points), with higher scores indicating better conditions.

The eTBL was calculated using the Gross equation [18]: eTBL (mL) = preoperative blood volume (PBV) × (preoperative Hct − postoperative Hct)/average Hct + allogeneic or autologous blood transfusion volume. HBL (mL) = eTBL − intraoperative bleeding volume.

The PBV was calculated using the method proposed by Nadler et al. [19]: PBV (mL) = k1 × height^3^ (m) + k2 × body weight (kg) + k3 (for men: k1 = 0.3669, k2 = 0.03219, and k3 = 0.6041; for women: k1 = 0.3561, k2 = 0.03308, and k3 = 0.1833).

### Surgical procedures

All surgeries were performed via the DAA. A three-dimensional porous titanium cementless cup (SQRUM TT; Kyocera Medical, Japan) and cementless tapered wedge femoral stems (J-Taper^TM^ HA stem or Initia stem; Kyocera Medical, Japan) were used in all cases. Intraoperative digital radiography was performed following a trial of acetabular and femoral component placement to check the implant size and position of fixation in all cases. ORC powder was applied intraoperatively to control bleeding from soft tissue and bone tissues, including the acetabular roof, after reaming, where it was difficult to coagulate and stop bleeding using an electric scalpel. 1000 mg of TXA was subfascially injected into the joint before wound closure in all cases. No surgical drains were used. The standard infection control protocols adhered to the guidelines of the Centers for Disease Control and Prevention. Patients were administered antibiotics, such as cefazolin, during surgery and on POD 1.

### Postoperative care interventions

For thrombosis prophylaxis, edoxaban was administered on the evening of POD1 and continued for 14 days. The dosage (15 or 30 mg) was determined based on the patient’s body weight or renal function. Additionally, mechanical thromboprophylaxis, including intermittent pneumatic compression therapy and elastic stockings, was implemented alongside anticoagulant therapy. Physiotherapy interventions, such as early postoperative ambulation and physical rehabilitation, were also administered to all patients. On POD 1, all the patients commenced full weight-bearing exercises.

### Statistical analyses

Statistical analyses were performed using GraphPad Prism version 10 (GraphPad Software, San Diego, CA, USA). Descriptive statistics were used to summarize the baseline characteristics and outcomes. Continuous variables were expressed as means and standard deviations (SDs). Student’s *t*-test and Fisher’s exact test were used to analyze quantitative and qualitative variables, respectively. Statistical significance was set at *p* < 0.05.

## Results

In total, 133 patients were included in this retrospective study. Table 1 summarizes the patient demographics and clinical characteristics. The mean patient age was 73.6 ± 8.4 years, and 85.0% (*n* = 113) were female. The mean BMI was 24.7 ± 3.9 kg/m^2^. Preoperatively, the mean hemoglobin (Hb) level was 12.3 ± 1.2 g/dL, and the mean hematocrit (Hct) was 37.5% ± 3.2%. No significant differences in patient demographics were observed between groups.

**Table 1:** Patient demographics and clinical characteristics.

	Total	Control group	ORC Powder group	*p*-value
	133 hips	80 hips	53 hips	
Age at operation (years)	73.6 ± 8.4	72.4 ± 9.1	75.1 ± 7.1	0.07
Sex (male/female)	20/113	15/65	5/48	0.11
Height (cm)	153.4 ± 8.0	153.5 ± 7.8	153.2 ± 8.4	0.84
Weight (kg)	58.4 ± 11.6	59.2 ± 11.9	57.3 ± 11.2	0.38
BMI (kg/m^2^)	24.7 ± 3.9	25.0 ± 4.2	24.3 ± 3.4	0.30
Crowe 1/2/3	100/18/15	63/ 9/ 8	37/ 9/ 7	
	75.2%/13.5%/11.3%	78.5%/11.3%/10%	69.8%/17.0%/13.2%	
ASA-PS 1, 2/3, 4	123/10	73/7	50/3	0.38

Values are expressed as the mean ± standard deviation. BMI, body mass index; ASA-PS, American Society of Anesthesiologists Physical Status; ORC, oxidized regenerated cellulose.

Table 2 summarizes perioperative hemorrhage parameters. There were no significant differences in the operative time or intraoperative blood loss between the two groups. The eTBL and HBL were both significantly lower in the ORC powder group (875.8 ± 292.9 mL vs. 679.1 ± 230.1 mL, *p* < 0.0001, 558.6 ± 264.2 mL vs. 424.1 ± 194.5 mL, *p* = 0.002, respectively). The number of patients who underwent autologous blood transfusion was 80.0% (64/80) in the control group and 28.3% (15/53) in the ORC powder group, with the control group showing a significantly higher rate. No allogeneic blood transfusions were administered in either group.

**Table 2:** Comparison of perioperative hemorrhage parameters between groups.

	Control group	ORC Powder group	*p*-value
	80 hips	53 hips	
Operating time (minutes)	101.7 ± 18.8	95.5 ± 15.5	0.06
Intraoperative bleeding (mL)	317.2 ± 184.1	255.0 ± 179.7	0.06
HBL (mL)	558.6 ± 264.2	424.1 ± 194.5	0.002
eTBL (mL)	875.8 ± 292.9	679.1 ± 230.1	<0.0001
Autologous blood transfusion rate (%)	80.0% (64/80)	28.3% (15/53)	<0.0001

Values are expressed as the mean ± standard deviation. HBL, hidden blood loss; eTBL, estimated total blood loss; ORC, oxidized regenerated cellulose.

Table 3 summarizes the trends in laboratory data and clinical outcomes before and after surgery. Postoperative Hb and Hct levels declined on POD 1, with partial recovery on POD 7. CRP levels returned toward baseline by POD 7, with no significant differences. The JOA score improved in both groups, and there was no significant difference between them. Postoperative pain, assessed using NRS, was significantly lower in the ORC powder group at POD 7 (2.9 ± 2.2 vs. 1.9 ± 1.6, *p* = 0.009).

**Table 3:** Trends in laboratory data and clinical outcomes before and after surgery.

	Control group	ORC powder group	*p*-value
	80 hips	53 hips	
Hemoglobin level (g/dL)			
Preoperative	12.3 ± 1.2	12.5 ± 1.2	0.45
POD1	10.4 ± 1.3	10.7 ± 1.1	0.19
POD7	10.7 ± 1.5	10.4 ± 1.1	0.12
Hematocrit level (%)			
Preoperative	37.5 ± 3.2	38.4 ± 3.3	0.14
POD1	31.5 ± 3.8	32.3 ± 3.1	0.17
POD7	32.5 ± 3.6	31.9 ± 3.3	0.32
C-reactive protein (mg/dL)			
Preoperative	0.29 ± 0.5	0.44 ± 0.9	0.21
POD7	1.3 ± 1.2	1.2 ± 1.2	0.53
Preoperative JOA score	45.8 ± 10.2	47.1 ± 7.9	0.45
Postoperative JOA score	85.9 ± 8.3	83.2 ± 6.6	0.07
Postoperative pain (NRS)			
POD7	2.9 ± 2.2	1.9 ± 1.6	0.009

Values are expressed as the mean ± standard deviation. POD, postoperative days; JOA score, Japanese Orthopedic Association score; NRS, numeric rating scale; ORC, oxidized regenerated cellulose.

The incidence of postoperative complications is shown in Table 4. The difference in the incidence of postoperative complications between the groups was not significant.

**Table 4:** Incidence of postoperative complications.

	Control group	ORC Powder group	*p*-value
	80 hips	53 hips	
Superficial infection	3.8% (3/80)	0	0.21
Deep infection	0	0	
Deep vein thrombosis	1.2% (1/80)	0	0.60
Symptomatic pulmonary embolism	0	0	
Revision surgery	0	0	

ORC, oxidized regenerated cellulose.

## Discussion

This retrospective study evaluated the efficacy of ORC powder in primary THA via the DAA. Our results demonstrated that patients treated with ORC powder had significantly lower eTBL and HBL compared to those who did not receive the intervention, without increasing the complication rate. Additionally, the ORC powder group exhibited reduced postoperative pain on POD7.

Various hemostatic agents have been utilized to control perioperative bleeding in THA, with TXA and ORC being commonly employed [10, 11, 20, 21]. The most prevalent method involves the intravenous or intra-articular administration of TXA, and its effectiveness in reducing perioperative blood loss in THA has been well-documented in numerous studies [10, 20]. The primary mechanism of action of TXA is the inhibition of the fibrinolytic system, suppressing its activation by preventing the conversion of plasminogen to plasmin. This mechanism inhibits fibrinolysis, making TXA a hemostatic agent associated with secondary coagulation, thereby facilitating hemostasis.

In contrast, the hemostatic effect of ORC differs from that of TXA. As a topical absorbable hemostatic agent, ORC is involved in primary coagulation and promotes clot formation when applied directly to the bleeding site. Made up of clusters of ORC fine fibers, SURGICEL^®^ Powder demonstrates enhanced in vitro clotting efficiency relative to its individual ORC fine fiber components, owing to its more advantageous surface energetics and increased surface area [14]. The fine powder form of the ORC allows easy and safe application in areas where hemostasis is challenging with thermal coagulation, such as acetabular bone tissue after reaming or bleeding from the branches of the obturator artery and surrounding vessels. Although TXA is associated with secondary coagulation, ORC is involved in primary coagulation. In this study, the combination of hemostatic agents with distinct mechanisms of action demonstrated superior hemostatic efficacy compared to that of TXA alone. In both groups, the JOA score improved favorably, with no significant differences in complication rates.

Several studies have investigated the role of ORC powder in reducing blood loss during orthopedic procedures, including THA. Wang et al. [12] reported that ORC powder application significantly reduced the perioperative blood loss in patients undergoing TKA, yielding favorable clinical outcomes. Similarly, Wakasa et al. [21] evaluated ORC powder use in THA and found no significant differences in eTBL, HBL, or clinical outcomes between the intravenous TXA + ORC powder and intravenous TXA + topical TXA groups. Their study differed from ours in terms of surgical approach, comparison of topical TXA and ORC powder, and the absence of postoperative anticoagulant drug therapy. However, our results are consistent with the finding that ORC powder contributes to a reduction in perioperative bleeding volume without reducing clinical outcomes in THA.

Interestingly, NRS scores on POD7 were significantly lower in the ORC powder group, because HBL is regarded as one of the causes of postoperative wound swelling [22], so reducing HBL is of high clinical importance. We hypothesized that the use of the ORC powder may have contributed to the reduction in postoperative thigh swelling by reducing HBL and eTBL. If hematoma formation was less frequent in the ORC powder group, it may reflect effective bleeding control, particularly in highly vascularized hip joint regions. Furthermore, compared with thermal coagulation hemostasis using an electric scalpel, hemostasis using ORC powder induces less tissue damage, which may offer potential benefits in infection control and minimizing tissue damage [23]. However, this study did not objectively evaluate postoperative thigh swelling, highlighting the need for further research to investigate this aspect.

A key strength of this study was that it clarified the effectiveness of ORC powder in perioperative blood management during THA via DAA, eliminating the confounding impact of the surgical approach on blood loss. Despite its strengths, this study has several limitations that should be considered when interpreting the results. First, the retrospective nature of the study and the relatively small sample size may have affected the reliability of the results. Second, because the study was conducted at a single institution, the findings may not be generalizable to other clinical settings or populations. Finally, strictly speaking, there is a possibility that the red blood cell content differs between MAP transfusion and autologous blood, and this cannot be ruled out as a factor influencing truly accurate eTBL calculations. In this study, there was a significantly higher rate of autologous blood storage in the control group, which may have affected the reliability of accurate eTBL calculations. However, this study used the same calculation formula as many previous studies.

In conclusion, the use of ORC powder during THA via the DAA can effectively reduce perioperative blood loss without increasing complication rates. These findings suggest that ORC powder has the potential to be a valuable adjunct in optimizing blood management strategies in THA.

## Data Availability

All data generated or analyzed during this study are included in this published article.
